# The Oriental Intruders in the Checklist: First European Records of Two Non-Native *Eumerus* Species (Diptera: Syrphidae) with Taxonomic Revisions of the European Fauna

**DOI:** 10.3390/insects17100981

**Published:** 2026-09-22

**Authors:** Ana S. Grković, Snežana Radenković, Martin Hauser, Tamara Tot, Jelena Ačanski, Marina Janković Milosavljević, Ante Vujić

**Affiliations:** 1 Department of Biology and Ecology, Faculty of Sciences, University of Novi Sad, 21000 Novi Sad, Serbia; snezana.radenkovic@dbe.uns.ac.rs (S.R.); tamarat@dbe.uns.ac.rs (T.T.); marinaj@dbe.uns.ac.rs (M.J.M.); ante.vujic@dbe.uns.ac.rs (A.V.); 2 Plant Pest Diagnostics Centre, California Department of Food and Agriculture, Sacramento, CA 95832-1448, USA; mhauser@cdfa.ca.gov; 3 BioSense Institute—Research Institute for Information Technologies in Biosystems, University of Novi Sad, 21000 Novi Sad, Serbia; acanski@biosense.rs

**Keywords:** hoverflies, Syrphidae, *Eumerus*, Merodontinii, taxonomic revision, synonymy, non-native species, endemism

## Abstract

The hoverfly genus *Eumerus* includes both vital native pollinators and potential agricultural pests, yet taxonomic ambiguities and missing distribution data hinder effective conservation and pest management. To resolve questionable taxa and compile an updated European inventory, we examined historical type specimens, reviewed literature, and evaluated species descriptions. We designate a lectotype for *Eumerus alpinus* Rondani, 1857, establish four new synonymies (*Eumerus aurofinis* Grković, Vujić, & Radenković, 2015, is a junior synonym of *E. rusticus* Sack, 1932; *E. lasiops* Rondani, 1857, is a junior synonym of *E. barbarus* (Coquebert, 1804); *E. alpinus* Rondani, 1857, is a junior synonym of *E. olivaceus* Loew, 1848; *E. nebrodesius* Rondani, 1868, is a junior synonym of *E. amoenus* Loew, 1848; exclude four doubtful taxa; and record two non-native Asian species (*E. aurifrons* (Wiedemann, 1824) and *E. figurans* Walker, 1859) in Europe for the first time. The updated checklist comprises 81 valid European species. Biogeographical analysis reveals peak species richness in the Mediterranean (65 species), whereas Macaronesia exhibits the highest rate of localised endemism at 75% (9 of 12 recorded species), followed by the Mediterranean (28%) and Pannonian (25%) regions. With over 60% of assessed native European *Eumerus* species threatened according to the IUCN Red List, this updated taxonomic framework provides an essential baseline for monitoring non-native pest introductions and guiding targeted conservation strategies for vulnerable endemic pollinators.

## Simple Summary

Hoverflies of the genus *Eumerus* are ecologically and economically important, functioning both as key wild pollinators and as potential agricultural pests whose larvae feed on plant roots and bulbs. However, effective conservation and pest management have long been obstructed by confused nomenclature, outdated literature, and incomplete distribution records. To resolve these issues, we re-examined historical literature, evaluated original descriptions, and critically reviewed type specimens across European museum collections. We updated the European species list by establishing four new synonymies, removing four doubtful taxa, and documenting the first European records for two non-native Asian species likely introduced through the international plant trade. The updated checklist confirms 81 valid *Eumerus* species in Europe. Geographically, species diversity peaks in the Mediterranean region, whereas Macaronesia exhibits the highest proportion of unique endemics. Because over 60% of native European *Eumerus* species are currently threatened with extinction, this revised classification establishes a reliable baseline to help agricultural authorities detect invasive pests and support conservation efforts for vulnerable pollinators.

## 1. Introduction

The genus *Eumerus* Meigen, 1822 (Diptera: Syrphidae; type species: *Syrphus tricolor* Fabricius, 1798) is one of the most species-rich hoverfly genera globally, comprising over 300 described species worldwide [1]. Prior to this study, 87 species were formally listed for the region [2]; however, by re-evaluating historical literature and type material, alongside documenting two Oriental species in Europe for the first time, the present work refines the valid European fauna to 81 species. The genus is primarily distributed across the Old World, with the highest diversity in the Mediterranean [3,4,5,6] and Afrotropical regions [7,8,9,10,11], while also comprising a remarkable number of species in Central Asia [12,13,14]. Larval stages of *Eumerus* species are primarily saprophytophagous, developing within bulbs, tubers, rhizomes, and stems of various plants [15,16,17,18,19,20,21]. This feeding behaviour has enabled the unintentional spread of several *Eumerus* species through global plant trade, resulting in their establishment beyond their native ranges [9,16,22]. Notably, *Eumerus narcissi* Smith, 1928 was even described from its newly established range, with its type locality recorded as Santa Cruz, California [16,23]. Speight et al. [16] were the first to report *E*. *narcissi* from Europe, documenting its presence in southern France. In their study, they also discussed the species’ introduction and establishment in North America, providing insights into its historical spread and association with the global trade of *Narcissus* bulbs, the primary host plants for its larvae. This species has been recently found in Corsica [24], Britain [25] and Italy [26]. In the Americas, at least five more *Eumerus* species have been recorded outside their native ranges, including *E*. *strigatus* (Fallén, 1817) and *E*. *funeralis* Meigen, 1822 [9,16,27,28,29,30,31], which are also introduced in Australia and New Zealand [32]. All three mentioned species belong to the *strigatus* group, a species group native to the Palearctic region. They primarily develop in the bulbs of *Narcissus* (Amaryllidaceae), onion, and other commercially cultivated plants and are already known to cause damage to crop fields, indicating their potential as agricultural pests [18,28,33,34,35,36,37,38,39,40,41,42,43,44,45,46].

On the other hand, the *obliquus* species group includes widespread Afrotropical and Oriental representatives. One of them, *Eumerus obliquus* (Fabricius, 1805), which is native to the Afrotropics, has been recorded in Brazil, Argentina, and Paraguay [9,22,31,47] and is also widespread in the Mediterranean, the Canary Islands [48,49,50], the Middle East and Australia and Oceania [9,51]. Its broad distribution is likely due to the ability of its larvae to develop in various plant tissues, ranging from *Opuntia* to commercially grown *Solanum* plants [16,20,52]. Another similar species, *Eumerus vestitus* Bezzi, 1912, has been reported from the Arabian Peninsula [53], India [54], and Pakistan [55]. In Europe, this species is recorded in Greece and Cyprus with scattered records. Considering its wide host preferences, Anooj et al. [54] highlight the potential of *E*. *vestitus* as a pest species for commercially grown plants. While there is a potential for certain *Eumerus* species to establish and spread in new environments, sometimes with economic consequences, many *Eumerus* species are rare and localised, with some facing conservation concerns due to habitat destruction and environmental changes [56]. This dual nature of the genus, comprising both potential pest species and vulnerable taxa, requires careful monitoring of newly introduced species. This is particularly evident in the European context, where the historical establishment of non-native elements is now joined by the arrival of species from entirely different biogeographical realms. In this study, we document for the first time the presence of two non-native species of Oriental origin in Europe: *Eumerus aurifrons* (Wiedemann, 1824) and *Eumerus figurans* Walker, 1859.

Consequently, the primary objectives of this study are to (1) document for the first time the presence of two non-native species of Oriental origin in Europe, *Eumerus aurifrons* (Wiedemann, 1824) and *Eumerus figurans* Walker, 1859; (2) provide a comprehensive, updated checklist of European *Eumerus* species; and (3) systematically resolve long-standing nomenclatural ambiguities by re-evaluating historical literature and type material. Ultimately, this work establishes a stable taxonomic and biogeographical framework essential for monitoring potential invasive alien pests and guiding targeted conservation planning for vulnerable endemic pollinators.

## 2. Materials and Methods

### 2.1. Studied Material and Collections

This study is based on the examination of adult specimens of *Eumerus* from several institutional and private collections. Material was studied directly or through high-resolution photographs when physical access was not possible. Photos of the holotype of *Eumerus figurans* are at the NMH, available at https://www.nhm.ac.uk/ (accessed on 15 May 2026). We examined material from the following collections:

FSUNS—Faculty of Sciences, University of Novi Sad, Novi Sad, Serbia;

MAegean—The Melissotheque of the Aegean, University of the Aegean, Mytilene, Greece;

M. H. coll.—Martin Hauser private collection;

M. B. coll.—Miroslav Barták private collection, Prague, Czech Republic;

MLUH—Center of Natural Science Collections of the Martin Luther University, Zoological Collection, Halle, Germany;

MZUF—Museo Zoologico della Università di Firenze, Florence, Italy;

MRSN—Museo Regionale di Scienze Naturali di Torino, Torino, Italy;

NMH—Natural History Museum, London;

SMF—Naturmuseum Senckenberg, Frankfurt, Germany;

ZMB—Museum für Naturkunde, Berlin, Germany;

ZMUC—Zoological Museum, University of Copenhagen, Denmark;

ZFMK—Zoological Research Museum Alexander Koenig, Bonn, Germany.

Specimen examination and morphological diagnoses were conducted using a Leica MZ16 stereomicroscope (Leica Microsystems GmbH, Wetzlar, Germany) under diffuse LED illumination. For specimens photographed by the authors, high-resolution photography was performed using a Canon EOS 6D Mark II camera (Canon Inc., Tokyo, Japan) body paired with a Canon MP-E 65 mm f/2.8 1–5× Macro Lens (Canon Inc., Tokyo, Japan). Lighting was supplied by two Canon Speedlite 430EX III-RT flash units (Canon Inc., Tokyo, Japan) controlled via a Canon Speedlite Transmitter ST-E2 (Canon Inc., Tokyo, Japan). Automated focus-stacking image series were captured using a Cognisys Stackshot Macro Rail Package (Cognisys Inc., Traverse City, MI, USA). Individual image Z-stacks were combined into composite images using Helicon Focus software (version 8.1.1), and improved in Adobe Photoshop.

### 2.2. Terminology

The morphological terminology used in the diagnoses is based on the terminology established by Thompson [57]. Terminology referring to male genitalia follows Doczkal [58]. Full label data are provided for type specimens. Label data are reproduced verbatim, with a slash (/) indicating a line break on the original label.

### 2.3. Species Groups

The classification of species into groups follows established literature [3,6,16,53,59,60,61]. However, certain modifications were made to ensure nomenclatural stability and priority:

*excisus* group: The previously used “*torsicus* group” is here referred to as the *excisus* group, as *E*. *excisus* Becker, 1921 holds priority over the more recently described *E*. *torsicus* Grković & Vujić, 2015.

*longicornis* group: For nomenclatural simplicity, the group formerly referred to as “*minotaurus*” is here designated the *longicornis* group, named after the senior synonym *E*. *longicornis* Loew, 1855. Furthermore, the name reflects the primary diagnostic character for the group: the distinctly elongated pedicel. The *minotaurus* complex is retained as a subordinate rank within this group to maintain consistency with recent revisions [4].

*clavatus* group: To ensure nomenclatural stability, the name *clavatus* group is retained here. Although *E*. *uncipes* Rondani, 1850, was described prior to *E*. *clavatus* Becker, 1921, the latter name is more widely established in current literature regarding this specific group’s definition [3].

### 2.4. Biogeographical Analysis and Diversity Metrics

Species richness and endemism patterns were analysed using the Biogeographical Regions of Europe map [62] as the spatial framework. Species were assigned to one or more regions based on verified occurrence data. To evaluate diversity, two metrics were employed: Total species richness was calculated as the absolute number of valid *Eumerus* species recorded within each biogeographical region. Local Endemism Rate: To distinguish between broad regional distributions and high-priority conservation targets, we applied a strict endemism criterion. Species were classified as “localised endemics” only if their known distribution is quantitatively restricted to a single discrete spatial unit defined as a single island or archipelago, a single isolated mountain range, or a single localised habitat patch (occupying ≤ 1 biogeographical sub-region or polygon). The endemism rate was calculated as the percentage of these localised endemics relative to the total species richness of that specific region.

### 2.5. Mapping

Distributional data for *Eumerus aurifrons* and *Eumerus figurans* were compiled from the online biodiversity database iNaturalist, examined specimens, material provided by the third author, and relevant literature sources. All records were visualised and mapped using QGIS. The species richness map was made in QGIS v3. × (QGIS Association, http://www.qgis.org (accessed on 15 June 2026)). Species richness data, derived from the compiled checklist and literature review, were integrated into the layer’s attribute table for each biogeographical region.

Occurrence data from iNaturalist (https://www.inaturalist.org (accessed on 5 June 2026)) were filtered to include European records of *Eumerus*, with species identifications individually verified by the authors.

Species distributions were summarised according to the biogeographical regions defined by the European Environment Agency (Figure 1) [62]. The following codes were used: Alpine—ALP, Anatolian—ANA, Arctic—ARC, Atlantic—ATL, Black Sea—BLS, Boreal—BOR, Continental—CON, Macaronesian—MAC, Mediterranean—MED, Pannonian Biogeographical Region—PAN, and Steppic—STE.

## 3. Results

### 3.1. New Records for European Fauna

**Subfamily Eristalinae Newman, 1834** [63]
  **Tribe Merodontini Edwards, 1915** [64]
  **Genus *Eumerus* Meigen, 1822** [65]

*Syrphus tricolor* Fabricius, 1798:563 [66], by subsequent designation of Curtis, 1839: 749 [67].

***Pumilio*** **Rondani, 1850: 127.**

*Pumilio delicatae* Rondani, 1850 (monotypy) [68].

***Citibaena*** **Walker, 1856: 124.**

*Citibaena aurata* Walker, 1856 (monotypy) [69].

***Citibena*** **Bigot, 1883: 225.**

Misspelling of *Citibaena* Walker [70].

***Paragopsis*** **Matsumura, 1916: 250.**

*Paragopsis griseofasciatus* Matsumura, 1916 (by original designation) [71].

***Microxylota*** **Jones, 1917: 230**.

*Microxylota robii* Jones, 1917 (by original designation) [72].

***Citabaena*** **Curran, 1938: 7.**

Misspelling of *Citibaena* Walker [73].

 

***Eumerus aurifrons* (Wiedemann, 1824)**

*Pipiza aurifrons* Wiedemann, 1824: 32. Type locality: “India orientali” [East India] [74].

*Eumerus albipes* Keiser, 1971: 302. Type locality: Madagascar [75].

**Figure 2, Figure 3 and Figure 4**

**Diagnosis**. Eyes dichoptic in both sexes, densely covered with distinctive yellow hairs (Figure 2A,C); tarsomeres 2–5 of hind leg broad and flattened in males, densely covered in short, snow-white hairs (Figure 2D); abdominal tergite 2 with a pair of large, luminescent pale-yellow spots, in addition to yellowish-white oblique bands on abdominal tergites 3 and 4 (Figure 2B). Male genitalia: Small-sized and stubby. Posterior surstyle lobe oval (Figure 3A,B). Hamus significantly enlarged (Figure 3C).

**Figure 2:**
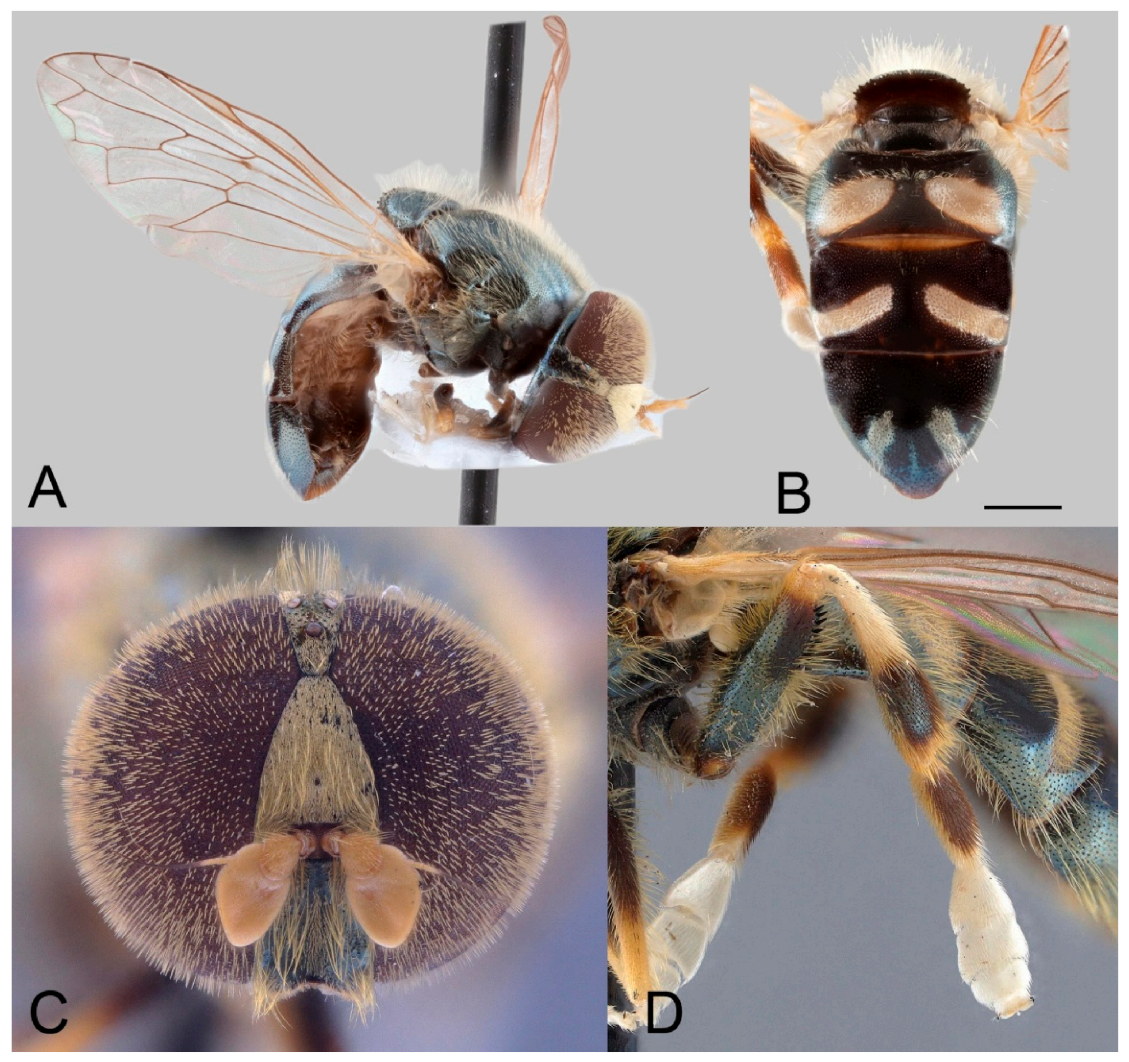
*Eumerus aurifrons*, male: (**A**,**B**) specimen from Palermo, Sicily; (**C**,**D**) specimen from Martin Hauser collection. (**A**) Habitus, lateral view; (**B**) abdomen, dorsal view; (**C**) head, anterior view; (**D**) hind leg, lateral view. Scale bars: 1 mm. (**A**,**B**) Photo: A. S. Grković. (**C**,**D**) Photo: M. Hauser.

**Figure 3:**
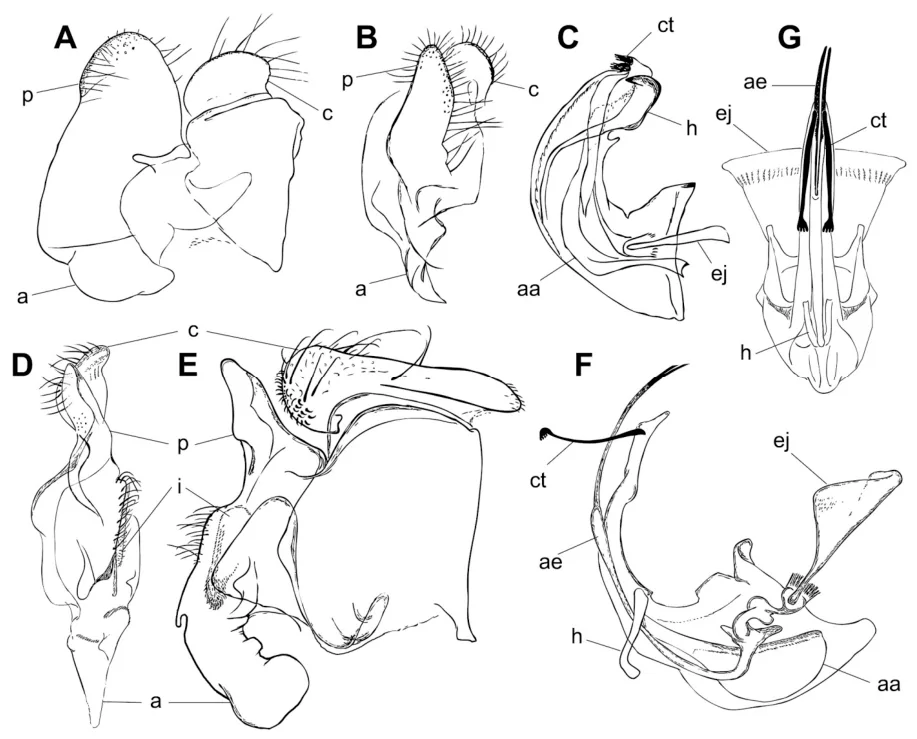
Male genitalia: (**A**–**C**) *Eumerus aurifrons*; (**D**–**G**) *Eumerus figurans*. (**A**) epandrium, lateral view; (**B**) epandrium, ventral view; (**C**) hypandrium, lateral view; (**D**) epandrium, ventral view; (**E**) epandrium, lateral view; (**F**) hypandrium, lateral view; (**G**) hypandrium, ventral view. Abbreviations: a—anterior surstyle lobe, aa—aedeagal apodeme, ae—aedeagus, c—cercus, ct—ctenidion, ej—ejaculatory apodeme, h—hamus, and p—posterior surstyle lobe.

**Examined material.** Mozambique: Maxixe, 1♂, 27.11–01.12.1997, leg. J. Bosák (ZFMK). Zimbabwe: Zambezi Valley, Rekomitjie, 4♂♂, 02.1987, leg. S. Gußmann (ZFMK). **New record.** Italy: Sicily, 20 km south of Palermo, 800 m, 1♂, 18.06.2012, leg. J. Halada (M. B. coll.).
**Distribution.** South and Southeast Asia, Africa, Australia, South America, Hawaii [9,51,74,76,77,78,79,80], and Italy (Sicily) (Figure 4).

**Figure 4:**
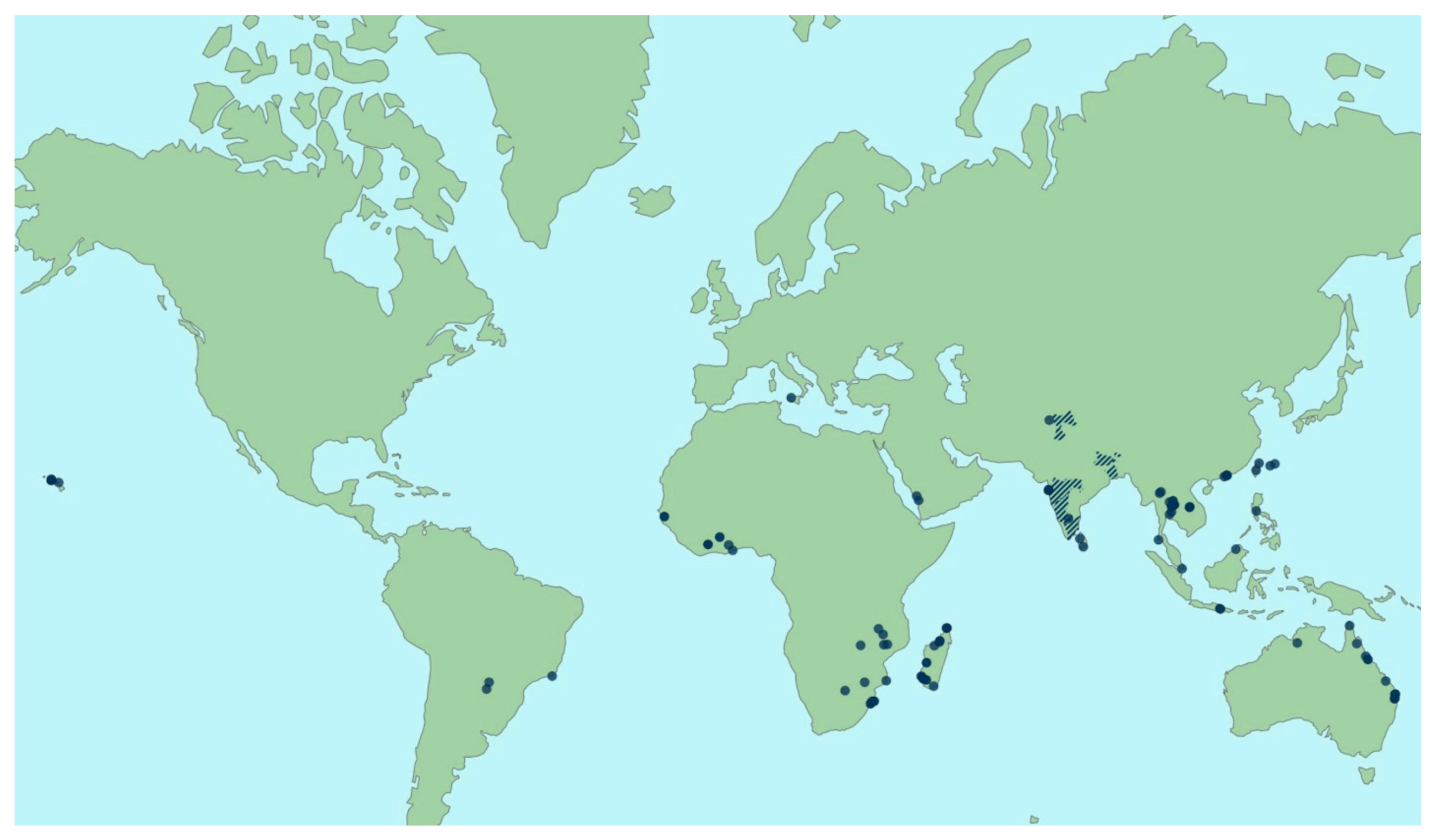
Distribution of *Eumerus aurifrons*. Precise record localities are indicated by dots; shaded areas correspond to regions reported by Mitra et al. [80] where no exact locality data are available.

 

***Eumerus figurans* Walker, 1859**
  *Eumerus figurans* Walker, 1859: 121. Type locality: Indonesia [81].
  *Eumerus marginatus* Grimshaw, 1902: 82. Type locality: Hawaii [82].
**Figure 3, Figure 5 and Figure 6**
  **Diagnosis.** Eyes almost bare; hind femur is more than three times as long as its maximum depth; scutellum rectangular, transversely elongated with posterior margin yellow; tergites black; dust spots on abdominal tergite 4 laterally sinuate (Figure 5). Male genitalia: Posterior surstyle lobe elongated, tapering apically (Figure 3D,E: p). Anterior surstyle lobe well developed (Figure 3D,E: a). Cercus elongated from lateral view (Figure 3D,E: c). Ctenidion extremely elongated (Figure 3F,G: ct). Apex of aedeagus elongated, protruding from the hypandrium (Figure 3F,G: ae).

**Figure 5:**
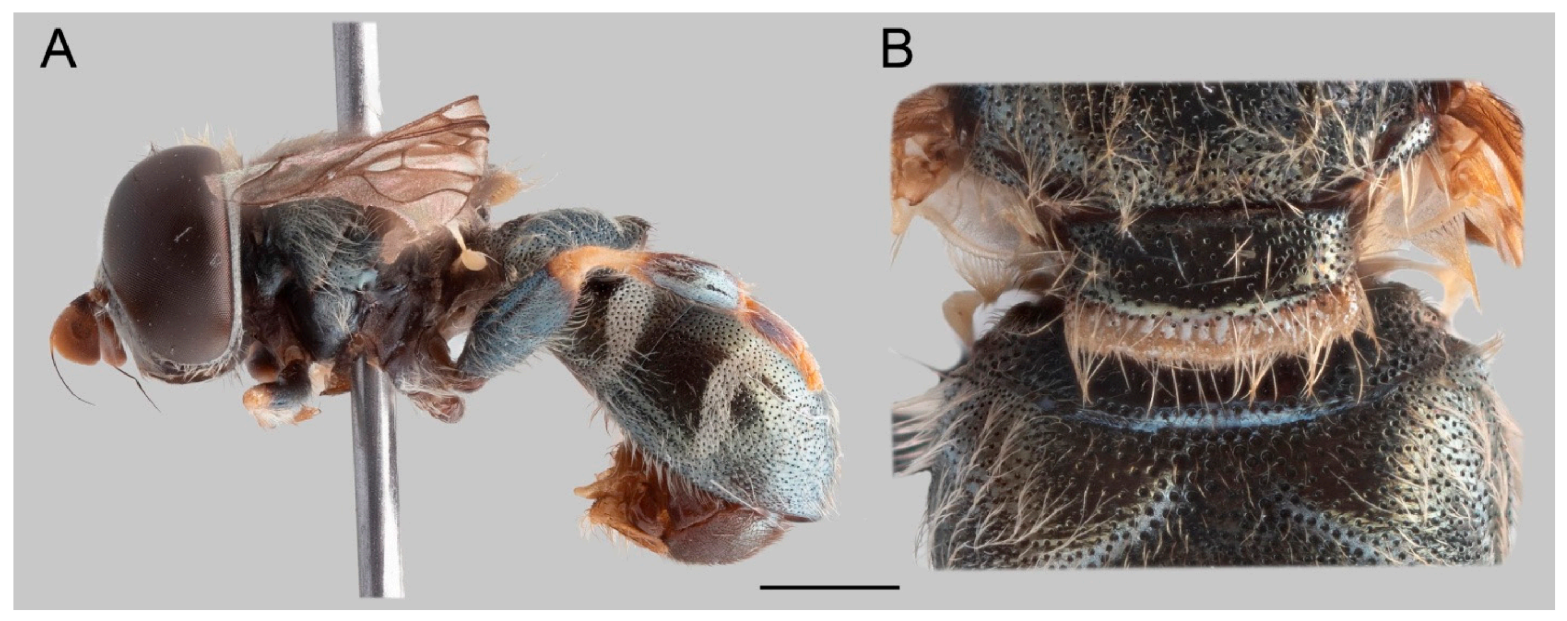
*Eumerus figurans*, male (Palermo, Sicily): (**A**) habitus, lateral view; (**B**) scutellum, dorsal view. Scale bar: 1 mm. Photo: A.S. Grković.

**Examined material. New record**. Italy: Sicily, 20 km south of Palermo, 800 m, 1♂, 18.06.2012, leg. J. Halada (M. B. coll.).
  **Remarks.** The identification is based on comparison with the original description and photographs of the holotype of *Eumerus figurans* Walker, 1859 housed in the Natural History Museum, London.
  **Distribution**. Oriental and Afrotropical regions (incl. Indonesia, Taiwan, Sri Lanka, India, SE Asia, Seychelles); Pacific: Hawaiian Islands [9,20,51,83]; Italy (Sicily) (Figure 6).
  **Note on distribution records.** Public data portal records (e.g., BOLD, GBIF; https://doi.org/10.15468/dl.78ufz6 (accessed on 4 March 2025)) indicating the presence of *Eumerus figurans* in Northwestern Australia were evaluated and excluded from our distribution data. Morphological examination of these Canadian National Collection (CNC) specimens (which lack the characteristic bicoloured scutellum with a yellow rim typical of true *E. figurans*) confirms that these records represent endemic Australian species and are the result of misidentifications.

**Figure 6:**
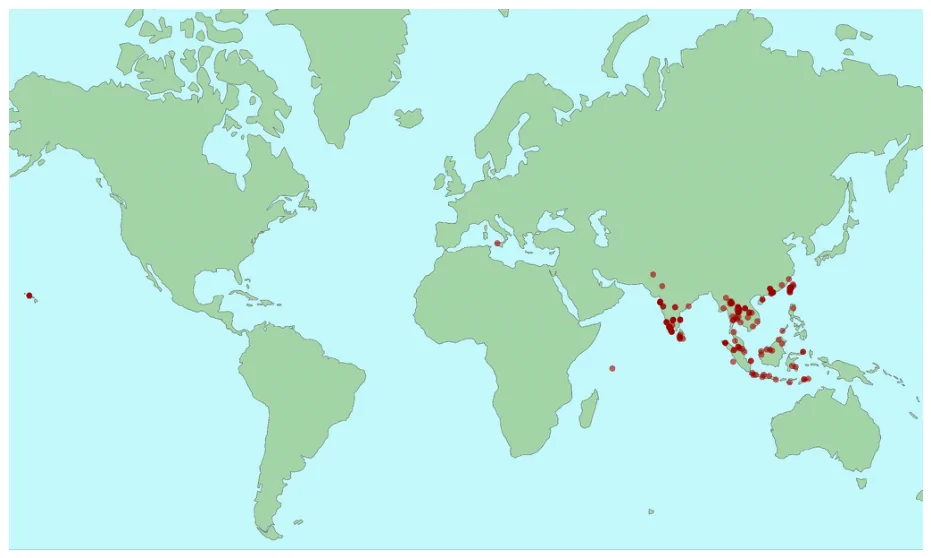
Distribution of *Eumerus figurans*. Precise record localities are indicated by dots.

### 3.2. Proposed Synonymy

***Eumerus amoenus*** **Loew, 1848**

  *Eumerus amoenus* Loew, 1848: 132. Type locality: (Sicily) Italy, Greece [84].

 

**=*Eumerus nebrodesius* Rondani, 1868: 575 syn. nov.** Type locality: (Sicily) Italy [85].

 

**Type material examined.** *Eumerus nebrodesius*. **MALE SYNTYPE.** [White handwritten label] “Madonie”; [white printed label] “148”; [red printed label, subsequent museum label] “Museo “La Specola”/coll. Rondani/HOLOTYPUS”; [white handwritten label] “nebrodesius/Rnd./Sicil.”; (MZUF).

**Notes**. *Eumerus nebrodesius* is listed as a valid species by Peck [23], Belcari et al. [86], Sforzi & Sommaggio [87]. After the examination of the male syntype preserved in the Museo “La Specola” (MZUF), we found it to be conspecific with *Eumerus amoenus*. Rondani [85] did not specify the number of specimens in his original description, making this specimen a syntype. The red “HOLOTYPUS” label was added subsequently during collection handling in the museum and does not constitute a valid holotype designation or lectotype fixation under ICZN rules.

 

***Eumerus barbarus*** **(Coquebert, 1804)**

  *Syrphus barbarus* Coquebert, 1804:117. Type locality: “Barbaria” [88].
    *Eumerus australis Meigen*, 1838:110. Type locality: Spain (Andalusia) [89].
    *Eumerus iris* Loew, 1848:118. Type locality: Italy (Sicily) [84].
    *Eumerus fucatus Rondani*, 1868:575. Type locality: Italy (Parma) [85].

**=*Eumerus lasiops* Rondani, 1857: 92 syn. nov.** Type locality: Italy (Parma) [90].

 

**Type material examined. FEMALE SYNTYPE.** [White printed label] “146”; [white label, partly handwritten] “Eumerus/barbarus Coq./(=E. lasiops Rnd.)/det. M. Daccordi 1994”; [red printed label] “Museo “La Specola”/coll. Rondani/SYNTYPUS”; [white printed label] “Museo “La Specola”/University of/Florence”; (MZUF).

 

**Note**. *Eumerus lasiops* Rondani, 1857, was described from Italy [90] based on both sexes, but the original description is inadequate for reliable recognition, lacking clear diagnostic characters. Peck [23] treated this species as of uncertain status, Dirickx [91] indicated that its validity requires confirmation, and Speight [2] noted that there is no basis for recognising it as a distinct species. Although Sforzi & Sommaggio [87] did not locate unambiguous type material during their survey, the female specimen from the Rondani collection in MZUF (box 4) examined here bears a museum “SYNTYPUS” label. Our direct examination of this specimen confirms it is conspecific with *Eumerus barbarus* (Coquebert, 1804) (confirming the previous determination by M. Daccordi in 1994). On this basis, *E. lasiops* is formally proposed as a junior synonym of *E. barbarus*.

 

***Eumerus olivaceus*** **Loew, 1848**

  *Eumerus olivaceus Loew*, 1848:116. Type locality: Italy (Sicily) [84].

 

**=*Eumerus alpinus* Rondani, 1857: 91**. Type locality: Italy (Piedmont) [90].

 

**Figure 7 and Figure 8**

**Type material examined.** *Eumerus olivaceus:* **MALE HOLOTYPE**. 1♂: [white handwritten label] “Sicilia/15 4 Zeller”; [red printed label] “Typus”; [white handwritten label] “*Eumerus*/*olivaceus*/m”; [white printed label] “Coll./H.Loew”; [white printed label] “Zool.Mus./Berlin”; [white printed label] “/http://coll.mfn-berlin.de/u/c2e416”; (ZMB) (Figure 7).

**Other material.** *Eumerus alpinus*: **MALE LECTOTYPE** (here designated in order to fix identity of the species). 1♂: [handwritten on small square yellow paper] “14.”; [printed in red on oval white paper] “131”; [red printed label] “Museo “La Specola”/coll. Rondani/SYNTYPUS” (Figure 8). **MALE PARALECTOTYPES**. 1♂: [printed in red on oval white paper] “131”; [red printed label] “Museo “La Specola”/coll. Rondani/SYNTYPUS” (MZUF); 4♂♂: [Specimen 1] [white printed label] “Eumerus/nudus Loew, 1848/Det. Sommaggio 2002 ♂”; [Specimen 2] bearing the same printed identification label; [Specimen 3] bearing the same printed identification label and a small, handwritten historical label “19”; [Specimen 4] bearing the same printed identification label and a handwritten historical label “28–68.” All four specimens share a collection box label: “E. alpinus Bell. ♂/Alpi” (MRSN).

**New material. Italy:** Belpasso, 5♂, 2♀, 06.04.2015, leg. A. Vujić et al. (FSUNS); Sicily, Pedara, 2♂, 1♀, 14.04.2024, leg. S. Radenković (FSUNS); 1♀, 14.04.2024, leg. T. Tot; 1♂ 18.04.2024, leg. S. Radenković, T. Tot; Ponte Antico Fiume Alcantara, Castiglione di Sicilia, 1♀, 20.04.2024, leg. S. Radenković (FSUNS).

**Figure 7:**
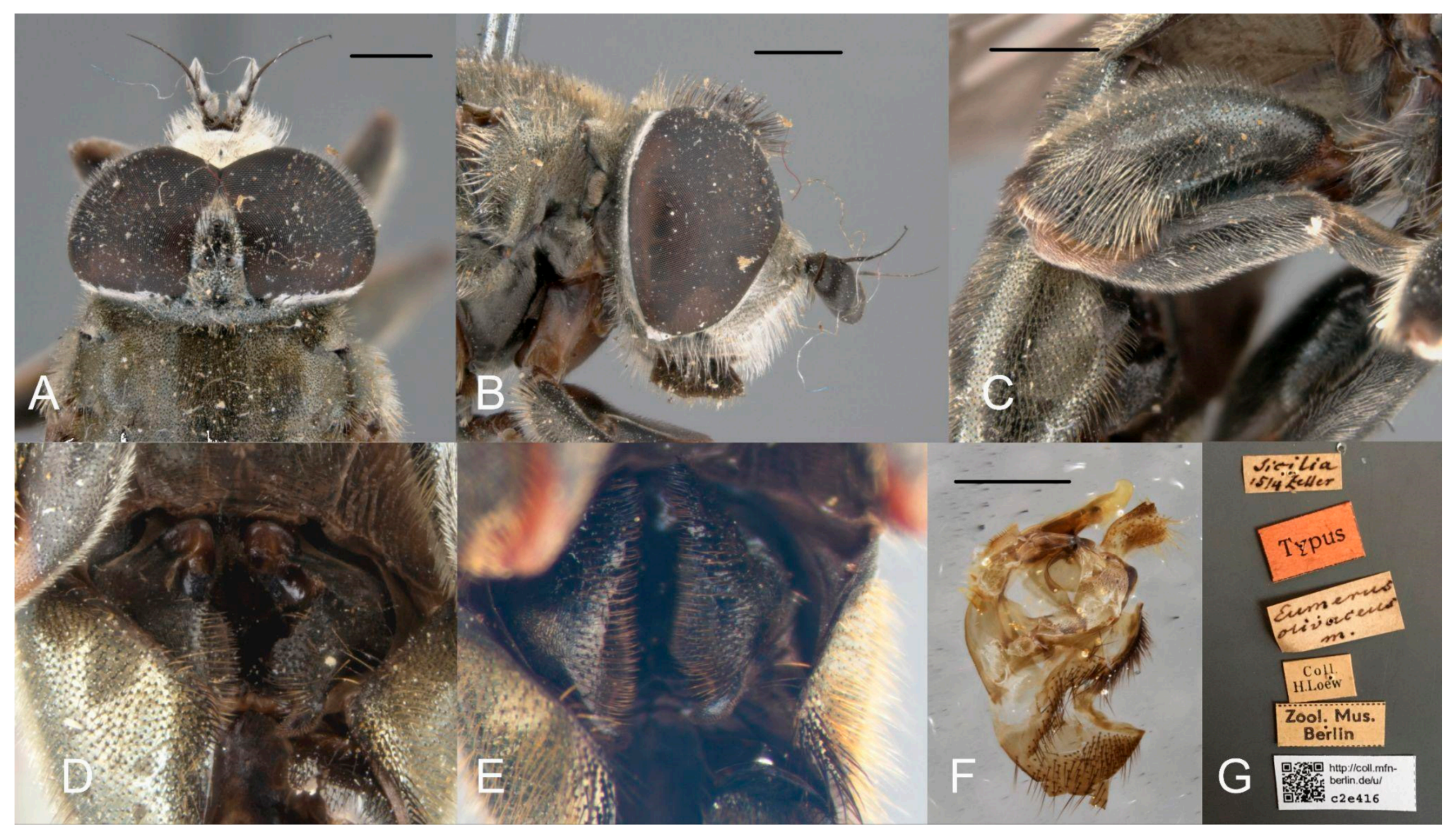
*Eumerus olivaceus* Loew, 1848: (**A**–**D**,**F**,**G**) male holotype (ZMB); (**E**) male from Montenegro. (**A**) head, dorsal view; (**B**) head, lateral view; (**C**) hind leg, lateral view; (**D**) sternite 4, showing symmetrical damage; (**E**) sternite 4 for comparison; (**F**) genitalia; (**G**) associated labels. Scale bars: 1 mm. (**A**–**D**,**F**,**G**) Photo: Eran Wolff, © Museum für Naturkunde Berlin; (**E**) Photo: A. S. Grković.

**Notes**. *Eumerus alpinus* was listed as a synonym of *E. olivaceus* by Peck ([23], p. 160), later resurrected as a valid species by Grković et al. ([92], p. 346), and is here resynonymised with *E. olivaceus*. The holotype of *Eumerus olivaceus* Loew, 1848, is housed in the Museum für Naturkunde, Berlin (ZMB) (Figure 7), while the syntype series of *E*. *alpinus* Rondani, 1857, is historically split between the MZUF (Florence) and the MRSN (Turin).

In the original description of *E*. *alpinus*, Rondani ([90], p. 91) stated: “Marem speciei in alpibus pedemontanis captum semel mihi Prof. Bellardi misit” [Prof. Bellardi sent me once a male of this species collected in the Piedmont Alps]. This phrasing led Grković et al. [92] to incorrectly conclude that only a single male specimen existed in the MZUF collection, which they treated as a holotype. However, Sforzi & Sommaggio [87] revealed that the original syntype series actually comprised six male specimens distributed across both the Florence and Turin collections.

To definitively fix the nomenclature and resolve longstanding ambiguities regarding this taxon, we evaluated the available syntype material. Our examination of the photographic data of the two MZUF syntypes confirmed that both specimens are morphologically conspecific and represent true *E*. *olivaceus*. The four remaining syntypes stored in the MRSN were previously identified by Sommaggio in 2002 as *E. nudus* Loew, 1848 [68]; however, based on the photos provided by the MRSN, these specimens belong to *E. olivaceus* (as *E. alpinus*) as well. To ensure nomenclatural stability, we herein designate the well-preserved male specimen from the Rondani collection in Florence (bearing the handwritten label “14.” and red collection number “131”) (Figure 8) as the lectotype of *Eumerus alpinus*. Consequently, the four MRSN specimens previously misidentified as *E. nudus* are here formally designated as paralectotypes of *E. alpinus*.

Grković et al. [92] previously resurrected *E*. *alpinus* as a valid species and restricted *E*. *olivaceus* as an endemic to Sicily. This decision was based on apparent morphological discrepancies between the *E*. *olivaceus* holotype (Figure 7) and continental European populations. However, our re-examination of the *E*. *olivaceus* holotype demonstrated that its sternite 4 was symmetrically damaged during the historical extraction of the male genitalia (Figure 7D), which led to a misinterpretation of its diagnostic characters.

Recent field sampling in Sicily yielded a larger series of *Eumerus* specimens from the topotypical region (Belpasso, Pedara, and Castiglione di Sicilia). A comparative analysis of this new topotypical material with the newly designated lectotype of *E*. *alpinus* confirms that they are morphologically identical. The structural differences previously used to split the two taxa are continuous variations or artefacts of preservation. Consequently, the identity of *E*. *olivaceus* is clarified: it is not an endemic island species, but rather a widely distributed taxon occurring across Southern Europe, from Greece to northern Spain, including the Apennine Peninsula and Sicily, with *E*. *alpinus* falling as its junior synonym.

**Figure 8:**
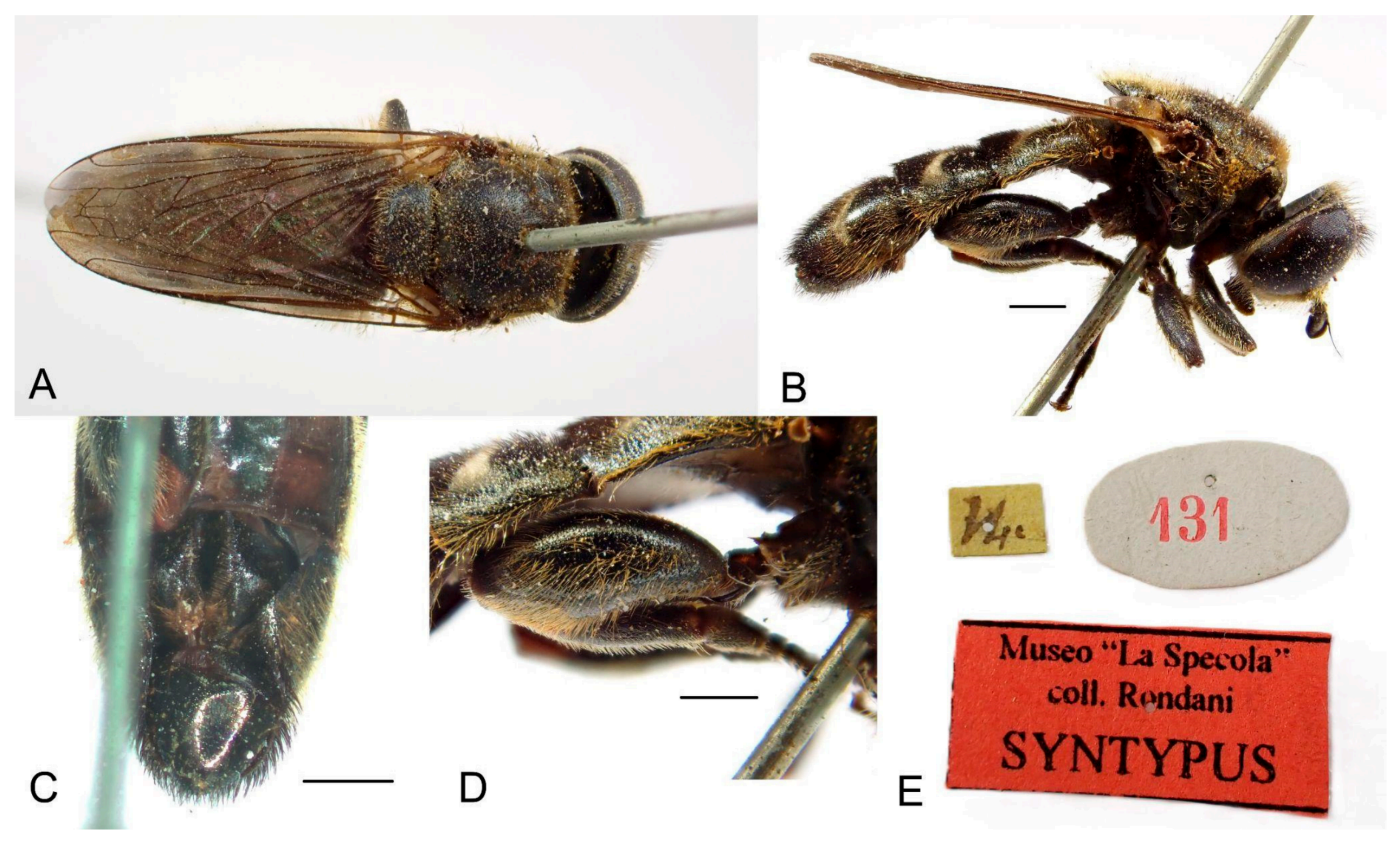
*Eumerus alpinus* Rondani, 1857, male lectotype (present designation) (MZUF): (**A**) habitus, dorsal view; (**B**) habitus, lateral view; (**C**) abdomen apex, lateral view; (**D**) hind leg, lateral view; (**E**) associated labels. Scale bars: 1 mm. Courtesy of the Museo di Storia Naturale dell’Università di Firenze (MZUF), Florence, Italy. Photo: G. F. Turrisi.

***Eumerus rusticus*** **Sack, 1932**

  *Eumerus rusticus* Sack, 1932:414. Type locality: Turkey (Asia minor) [93].

 

**=*Eumerus aurofinis* Grković, Vujić, & Radenković, 2015: 364 syn. nov.** Type locality: Greece (Samos) [92].

 

**Figure 9, Figure 10 and Figure 11**

**Examined material. Type material. MALE SYNTYPE.** [White label, partly handwritten] “Zool. Inst. Halle-S./Eumerus ♂/rusticus Sack/ det. P. Sack 1930”; [yellow handwritten label] “Typus”; [white handwritten label] “110”; [white printed label] “v. Röder”; [white handwritten label] “Asia min”; (MLUH) (Figure 9). **FEMALE SYNTYPE.** [White label, partly handwritten] “Zool. Inst. Halle-S./Eumerus ♀/rusticus Sack/ det. P. Sack 1930”; [yellow handwritten label] “Typus ♀”; [white printed label] “v. Röder”; [white handwritten label] “Asia min”; (MLUH) (Figure 10).

**Other material.** [White handwritten label] “Eumerus ♂/rusticus Sack”; [white handwritten label] “Asia min”; [white handwritten label] “Smyrna”; [white printed label] “v. Röder”; (SMF) (Figure 11).

**Figure 9:**
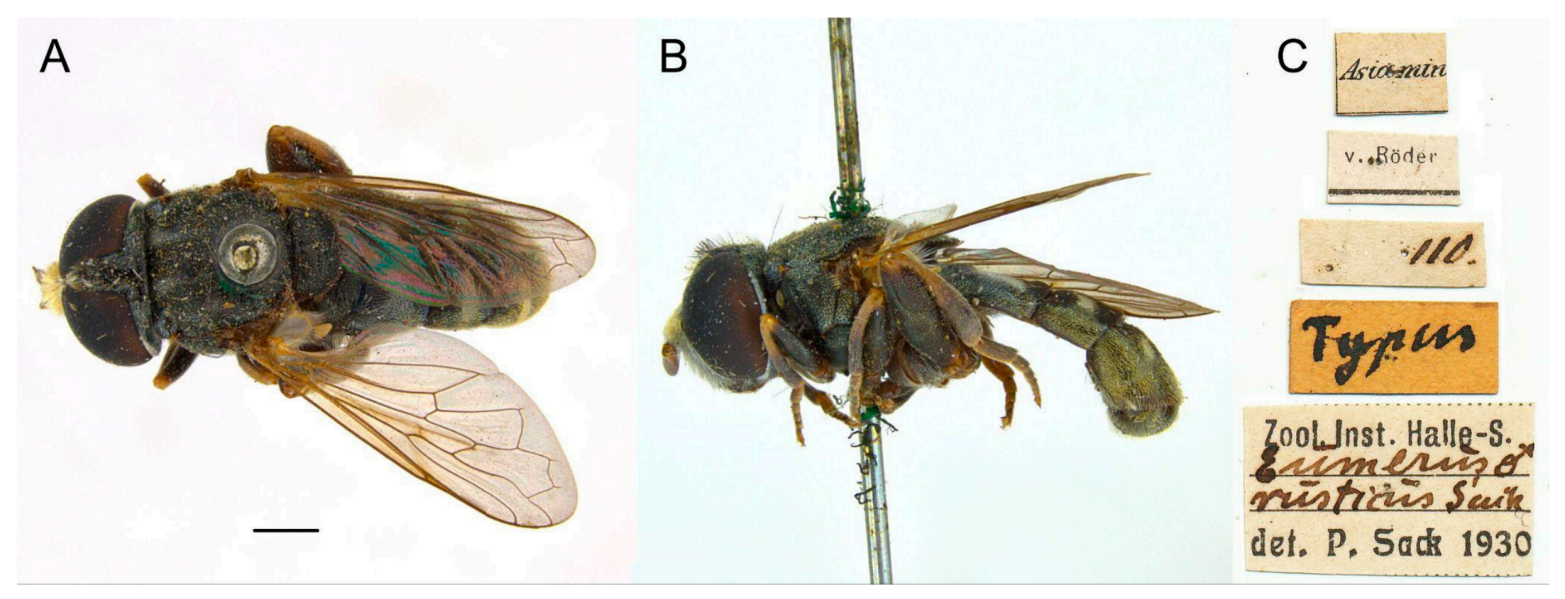
*Eumerus rusticus* Sack, 1932, male syntype (MLUH): (**A**) habitus, dorsal view; (**B**) habitus, lateral view; (**C**) associated labels. Scale bar: 5 mm. Courtesy of the Center of Natural Science Collections of the Martin Luther University, Zoological Collection, Halle, Germany. Photo: Joachim Händel.

**Figure 10:**
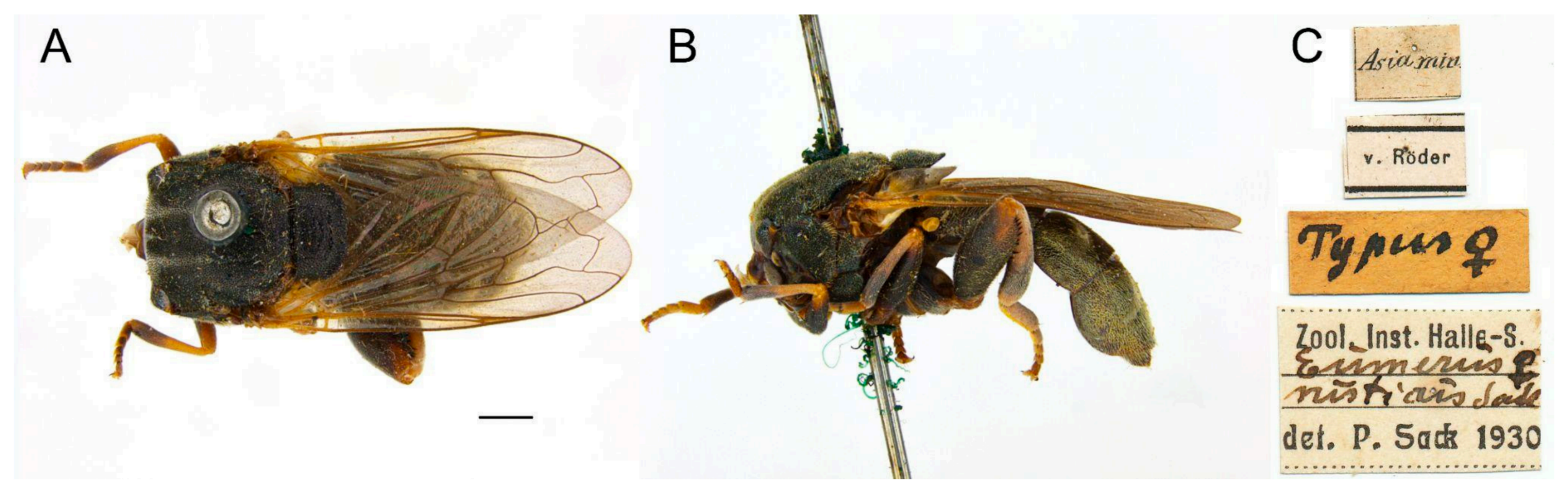
*Eumerus rusticus* Sack, 1932, female syntype (MLUH): (**A**) habitus, dorsal view; (**B**) habitus, lateral view; (**C**) associated labels. Scale bar: 1 mm. Courtesy of the Center of Natural Science Collections of the Martin Luther University, Zoological Collection, Halle, Germany. Photo: Joachim Händel.

**Notes**. The identity of *Eumerus rusticus* Sack, 1932 was clarified and confirmed through the examination of high-resolution photographs of the male and female syntypes deposited in the Martin Luther University Halle-Wittenberg (MLUH) (Figure 9 and Figure 10) and one historical male specimen from the von Röder collection from Naturmuseum Senckenberg, Frankfurt (SMF) (Figure 11). All of these specimens perfectly match the original description by Sack [93] as well as the description of *Eumerus aurofinis* Grković, Vujić, & Radenković, 2015 [92]. The species can be distinguished from related taxa of the *E. tricolor* group by the following diagnostic characters: Body covered in very short hairs, characteristic short golden hairs present on the top of abdomen; scutum black and dull with two triangular dust spots anteriorly; wing with brownish tinge; legs predominantly black, with the basal third to half of the tibiae, tips of the femora and ventral sides of the tarsi yellow; coxae and trochanters mostly black; hind femur very strong and thickened and dorsally convex; ventral subapical setae long and sharp, with 9–10 setae in the anterior row, located on a raised edge, and 6–7 setae in the posterior row; abdomen elongated and entirely black; pairs of white dust spots present on tergites 2 to 4; sternites simple, bronze to brown coloured and covered in short white hairs. In addition, molecular data (COI sequences) from Turkish specimens collected on Bozdağ Mountain, alongside Greek specimens from the islands of Samos and Rhodes, demonstrate that all analysed material of *Eumerus aurofinis* belongs to the same taxon [92].

*Eumerus aurofinis* was described based on material collected on the East Aegean Islands (Samos and Lesvos) and Turkey (Mountain Bozdağ). The primary type material (male holotype and paratypes) is deposited in FSUNS collection, where it was designated and directly examined by the authors during the original description [92].

Taking all morphological, geographical, and molecular evidence into account, *E. aurofinis* is here considered a junior synonym of *E. rusticus*.

**Figure 11:**
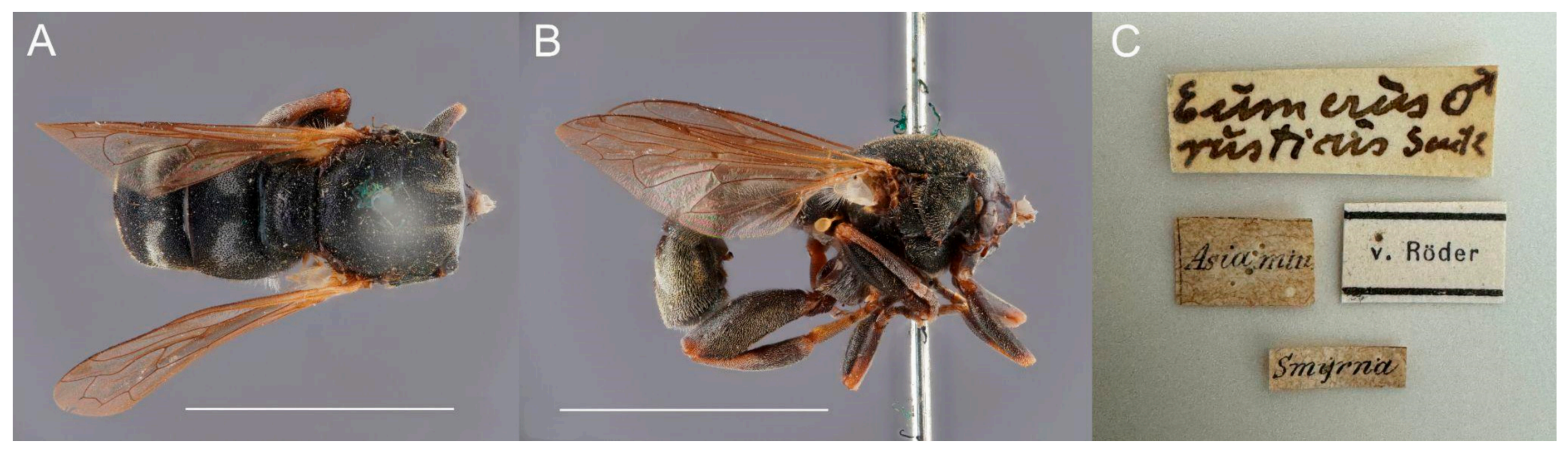
*Eumerus rusticus*, historical male specimen from the von Röder collection (SMF): (**A**) habitus, dorsal view; (**B**) habitus, lateral view; (**C**) associated labels. Scale bar: 5 mm. Courtesy of the Senckenberg Research Institute and Natural History Museum Frankfurt, Germany. Photo: Nikolai Ignatev. © Senckenberg.

### 3.3. Taxonomic Status of Eumerus pauper Becker, 1921

***Eumerus pauper*** **Becker, 1921**

*Eumerus pauper* Becker, 1921: 71. Type locality: Spain [94].

**=*Eumerus bifurcatus*** van Steenis & Hauser, 2019: Aguado-Aranda et al. (2025: 21) (as synonym). Type locality: Spain [13].
  **Type material.** The type material of *E*. *pauper* Becker, 1921 (ZMB) is currently unavailable for study; it has been on long-term loan since 1999 and remains unreturned despite the museum’s recovery efforts (J. Pohl, pers. comm.). The species was originally described from two female syntypes from Spain without a precise locality. A male was subsequently described by Gil Collado [95] based on additional Spanish material.
  **Remarks.** Aguado-Aranda et al. [96] examined historical material determined as *E*. *pauper* by Gil Collado (4♂♂, 2♀♀). Based on these specimens and the original descriptions, they concluded that *E*. *bifurcatus* van Steenis & Hauser, 2019, is identical to *E*. *pauper*. Notably, these authors attributed the drawings of the male genitalia from the original description of *E*. *bifurcatus* [13] to *E*. *pauper*. Although they did not formally propose a new synonymy (syn. nov.) due to the unavailability of the Becker types, they explicitly treated *E*. *bifurcatus* as a junior synonym by replacing its use with *E*. *pauper*. This interpretation is consistent with earlier expert feedback communicated to the last author (Claus Claussen, pers. comm.). Following this taxonomic judgment, the species are treated as synonyms here.

### 3.4. Checklist of Eumerus Species in Europe

The European fauna of the genus *Eumerus* currently comprises 81 valid species (Table 1). Two species, *Eumerus aurifrons* and *E*. *figurans* are newly recorded from Europe.

 

The taxonomic structure of the European fauna is distributed across 11 recognised species groups, while 10 species (12.3%) remain currently unassigned to any specific group or complex. The *tricolor* group represents the most species-rich group in the region, containing 23 species, followed by the *strigatus* group with 14 species.

 

The checklist below presents the currently valid *Eumerus* species known from Europe, based on literature records and recent revisions, including new data reported in this study.

**Table 1:** Taxonomic checklist of European *Eumerus* species with species group affiliation (including subgroups/complexes) and geographic distribution. Abbreviations: Alpine—ALP, Anatolian—ANA, Atlantic—ATL, Boreal—BOR, Continental—CON, Macaronesian—MAC, Mediterranean—MED, Pannonian Biogeographical Region—PAN, and Steppic—STE.

*Species*	*Species Group/Subgroup (Complex)*	*Distribution*
*Eumerus amoenus* Loew, 1848	*strigatus*	CON, MAC, MED, PAN
*Eumerus ancylostylus* Aguado-Aranda & Ricarte, 2023	*tricolor*	MED
*Eumerus arctus* van Steenis, 2021	*tricolor*	ALP (Switzerland)
*Eumerus argyropus* Loew, 1848	*ornatus*	MED, CON
*Eumerus armatus* Ricarte & Rotheray, 2012	*tricolor*	MED
*Eumerus aurifrons* (Wiedemann, 1824)	*Unassigned*	MED (Italy: Sicily)
*Eumerus azabense* Ricarte & Marcos-García, 2018	*tricolor*	MED
*Eumerus banaticus* Nedeljković, Grković, & Vujić, 2019	*strigatus/bactrianus*	PAN (Serbia, Romania)
*Eumerus barbarus* (Coquebert, 1804)	*barbarus*	MED
*Eumerus basalis* Loew, 1848	*basalis*	MED, CON
*Eumerus bayardi* Séguy, 1961	*tricolor*	MED
*Eumerus bicornis* Grković, Vujić, & Hayat, 2019	*strigatus/bactrianus*	MED, ANA
*Eumerus canariensis* Baez, 1982	*barbarus*	MAC
*Eumerus claripennis* Coe, 1957	*basalis*	MED
*Eumerus clavatus* Becker, 1923	*clavatus*	MED, CON, ANA
*Eumerus colladoi* Ricarte & Aguado-Aranda, 2022	*Unassigned*	MED
*Eumerus consimilis* Šimić & Vujić, 1996	*strigatus*	ATL, CON, MED, PAN
*Eumerus crassus* Grković, Vujić, & Radenković, 2016	*longicornis*	MED
*Eumerus crispus* Vujić & Grković, 2021	*tricolor*	PAN (Serbia)
*Eumerus dubius* Baez, 1982	*Unassigned*	MAC
*Eumerus emarginatus* Loew, 1848	*pulchellus*	MED
*Eumerus etnensis* van der Goot, 1964	*obliquus*	MED
*Eumerus excisus* van der Goot, 1968	*excisus*	MED (France: Corse)
*Eumerus figurans* Walker, 1859	*Unassigned*	MED (Italy: Sicily)
*Eumerus flavitarsis* Zetterstedt, 1843	*ornatus*	MED, CON, PAN
*Eumerus funeralis* Meigen, 1822	*strigatus*	ATL, CON, MED, PAN
*Eumerus gibbosus* van Steenis, Hauser, & van Zuijen, 2017	*barbarus*	MED
*Eumerus grallator* Smit, 2019	*tricolor/binominatus*	MED (Spain)
*Eumerus grandis* Meigen, 1822	*tricolor*	MED, CON, ALP, ATL
*Eumerus hispanicus* van der Goot, 1966	*tricolor*	MED
*Eumerus hispidus* Smit, Franquinho-Aguiar, & Wakeham-Dawson, 2004	*Unassigned*	MAC
*Eumerus hungaricus* Szilády, 1940	*Unassigned*	ALP, MED, CON
*Eumerus incisus* Malidžan & Vujić, 2022	*excisus*	MED (Montenegro)
*Eumerus karyates* Chroni, Grković, & Vujić, 2018	*longicornis/minotaurus*	MED (Greece: Peloponnese)
*Eumerus larvatus* Aracil, Grković, & Pérez-Bañón, 2022	*tricolor*	MED (Spain)
*Eumerus latitarsis* Macquart in Webb & Berthelot, 1839	*Unassigned*	MAC
*Eumerus longicornis* Loew, 1855	*longicornis*	CON, PAN
*Eumerus lucidus* Loew, 1848	*ornatus*	MED
*Eumerus macarius* Ricarte & Aguado-Aranda, 2025	*Unassigned*	MAC
*Eumerus minotaurus* Claussen & Lucas, 1988	*longicornis/minotaurus*	MED (Greece: Crete)
*Eumerus montanum* Grković, Radenković, & Vujić, 2017	*strigatus*	ALP, MED
*Eumerus narcissi* Smith, 1928	*strigatus*	ATL, MED, CON
*Eumerus niehuisi* Doczkal, 1996	*longicornis*	MED
*Eumerus nigrorufus* Grković & Vujić, 2021	*tricolor*	ALP, MED
*Eumerus nivariae* Baez, 1982	*Unassigned*	MAC
*Eumerus niveitibia* Becker, 1921	*tricolor*	MED
*Eumerus nudus* Loew, 1848	*olivaceus*	MED
*Eumerus obliquus* (Fabricius, 1805)	*obliquus*	MED, MAC
*Eumerus olivaceus* Loew, 1848	*olivaceus*	ALP, MED
*Eumerus ornatus* Meigen, 1822	*ornatus*	ALP, BOR, CON, MED, PAN
*Eumerus ovatus* Loew, 1848	*tricolor*	ALP, BOR, CON, MED, PAN
*Eumerus pannonicus* Ricarte, Vujić, & Radenković, 2016	*strigatus/bactrianus*	PAN (Serbia)
*Eumerus pauper* Becker, 1921	*strigatus/bactrianus*	MED
*Eumerus petrarum* Aguado-Aranda, Nedeljković, & Ricarte, 2023	*tricolor*	MED (Spain)
*Eumerus phaeacus* Loew, 1848	*longicornis/minotaurus*	MED
*Eumerus pulchellus* Loew, 1848	*pulchellus*	CON, MED, MAC
*Eumerus purpurariae* Baez, 1982	*obliquus*	MAC
*Eumerus purpureus* Macquart, in Webb & Berthelot, 1839	*Unassigned*	MAC
*Eumerus pusillus* Loew, 1848	*basalis*	CON, MED
*Eumerus richteri* Stackelberg, 1960	*tricolor*	CON, MED
*Eumerus rubrum* Grković & Vujić, 2017	*tricolor*	MED (Greece)
*Eumerus ruficornis* Meigen, 1822	*strigatus*	ALP, BOR, CON
*Eumerus rusticus* Sack, 1932	*tricolor*	MED
*Eumerus sabulonum* (Fallén, 1817)	*tricolor*	ALP, BOR, CON, MED
*Eumerus santosabreui* Baez, 1982	*pulchellus*	MAC
*Eumerus sardus* Aguado-Aranda, Ricarte, & Hauser, 2024	*barbarus*	MED
*Eumerus sicilianus* van der Goot, 1964	*obliquus*	MED (Italy: Sicily)
*Eumerus sinuatus* Loew, 1855	*tricolor*	ALP, CON, MED
*Eumerus sogdianus* Stackelberg, 1952	*strigatus*	ALP, BOR, CON, MED, STE
*Eumerus strigatus* (Fallén, 1817)	*strigatus*	ALP, ATL, BOR, CON, MED, STE
*Eumerus subornatus* Claussen, 1989	*ornatus*	MED
*Eumerus sulcitibius* Rondani, 1868	*barbarus*	MED
*Eumerus tarsalis* Loew, 1848	*tricolor*	ALP, BOR, CON, MED, STE
*Eumerus tauricus* Stackelberg, 1952	*tricolor*	CON, PAN, STE
*Eumerus tenuitarsis* Grković & Vujić, 2019	*tricolor/binominatus*	MED
*Eumerus torsicus* Grković & Vujić, 2015	*excisus*	MED
*Eumerus tricolor* (Fabricius, 1798)	*tricolor*	ALP, BOR, CON, MED, PAN, STE
*Eumerus truncatus* Rondani, 1868	*strigatus*	MED
*Eumerus uncipes* Rondani, 1850	*clavatus*	MED, CON
*Eumerus vandenberghei* Doczkal, 1996	*strigatus*	MED (France: Corsica)
*Eumerus vestitus* Bezzi, 1912	*obliquus*	MED

### 3.5. Species of Uncertain Status in the European Fauna

The species listed below are either of uncertain taxonomic identity, of doubtful occurrence in Europe, or both.

 

***Eumerus acuticornis*** **Sack, 1933**
      *Eumerus acuticornis* Sack, 1933:8. Type locality: North China [97].
     

*Eumerus acuticornis* was originally described from North China and is currently considered to have a Central to East Asian distribution. Its occurrence in Europe is doubtful. The only European record originates from Romania [98], but this record was already regarded as requiring confirmation by Dirickx [91]. No additional European material has been documented since, and no verified specimens are known from European collections.
  Gilasian et al. [99] suggested that *Eumerus acuticornis* is likely endemic to southern Mongolia. Evenhuis & Pape [100] listed *E. acuticornis* for Mongolia, supporting an Asian distribution pattern. In the absence of confirmed European material, *E. acuticornis* is here treated as a doubtful species for the European fauna.

 

***Eumerus angustifrons*** **Loew, 1848**
    *Eumerus angustifrons* Loew, 1848:125. Type locality: Turkey (Antalya) [84].
     

**Type material examined.** [White handwritten label] Adalia/Oct. 40.; [green handwritten label] Kl.Asien/Adalia/H. Loew; [red printed label] Typus; [white handwritten label] Eumerus/angustifrons/m.; [white printed label] Coll./H.Loew; [white printed label] MfN/URI/ http://coll.mfn-berlin.de/u/0ee7bb; (ZMB).

 

*Eumerus angustifrons* Loew, 1848, was described from Turkey (“Adalia”, modern-day Antalya). The female type specimen deposited in the Museum für Naturkunde, Berlin (Loew collection, ZMB) was directly examined. While this specimen is undeniably Loew’s type, its exact taxonomic identity, specifically whether it is conspecific with or distinct from other described species, remains uncertain. Because the type is a single female with a glued head, its key morphological characters cannot be evaluated with sufficient confidence to determine potential synonymy without topotypical male specimens.
  Direct examination of the female type specimen of *E. angustifrons* reveals the following morphological characters: The head is glued; the third antennal segment appears relatively large and rectangular, with a darkened dorsal and anterior margin and reddish colouration ventrally. The face is faintly dusted and white-haired; the frons is shiny, with only a narrow dusted stripe along the eye margin extending to the anterior ocellus. The ocellar triangle is slightly isosceles; hairs around the ocelli are black, with several additional black hairs on the vertex. Eyes covered in short, pale hairs. The hind trochanter angular ventrally. The hind femur is strong, with two rows of spines apicoventrally. The first row of sharp spines is normally developed, and the second row has only a few blunt-tipped spines. At the base of the ventral side of the hind femur, a small bulge is present, similar to that in *E. funeralis* Meigen, 1822.
  The species was described from Turkey. The Romanian record is based on Kowarz [101], Thalhammer [102], and Brădescu [98], but requires confirmation.

 

*Eumerus angustifrons* is listed as a valid species by Kowarz [101], Thalhammer [102], Sack [93], Peck [23], Brădescu [98], and Speight [2]. Dirickx [91] stated that the only records from Romania are those of Kowarz [101] and Thalhammer [102]. Evenhuis & Pape [100] listed this species as valid with a range in Turkey. Stackelberg [12] briefly referenced this species in a footnote, placing it in the same group as *E*. *chrysopygus* Sack, 1941 from China.

 

***Eumerus graecus*** **Becker, 1921**
  *Eumerus graecus* Becker, 1921:69. Type locality: Greece (Poros) [94].
     

The type material (3 syntypes: 2♂ and 1♀) of *Eumerus graecus* Becker, 1921, housed in the ZMB, is currently unavailable for study due to a long-term loan since 1999, despite the museum’s efforts to have it returned (Jenny Pohl, pers. comm.). Based on examination of comparative material from Greece that fits with the original description, as well as assessment of historically identified specimens (Martin Ebejer, pers. comm.), the diagnostic characters attributed to *E. graecus* appear to fall within the known range of intraspecific variation in the widely distributed Mediterranean species *E. pusillus* Loew, 1848. However, in the absence of direct examination of the type material, the taxonomic status of *E. graecus* cannot be confirmed with certainty. It is therefore treated as a doubtful species pending further study of the type material.

 

***Eumerus lunatus*** **(Fabricius, 1794)**

*Syrphus lunatus* Fabricius, 1794: 296. Type locality: “Barbaria” [103].

*Milesia lunata* (Fabricius, 1794): Fabricius, 1805: 193 (generic transfer) [104].

 

**Type material examined.** *Syrphus lunatus*: **Male holotype.** [White handwritten label] lunatus; [yellow handwritten label] 193.22; [red printed label] Type; [white printed label] NHMD/308691; (ZMUC).

**Other material.** *Milesia lunata*: **Male.** [red printed subsequent museum label] Type; [green handwritten label] Barb. (F.) MFW; [green handwritten label] Lunata F*W.; [purple printed label] 946; [yellow printed label] Zool. Mus./Berlin; [green handwritten label] Barbaria; (ZMB).

 

The taxonomic identity and European occurrence of *Eumerus lunatus* (Fabricius, 1794) remain unresolved. Smit et al. [53], in their revision of *Eumerus* from the Arabian Peninsula, associated *E*. *lunatus* with *E*. *lacertosus* Smit, 2017 and *E*. *triangularis* Hervé-Bazin, 1913, based on a set of shared characters without examination of the type material of *E*. *lunatus*.
  Subsequent studies [11] expanded the concept of the *E*. *triangularis* species group, whose distribution is restricted to the Afrotropical region, by identifying additional African members and refining its diagnosis. Furthermore, they emphasised that, based on the original description and examination of the remaining parts of the holotype, *E*. *lunatus* does not fit the diagnostic character set of the *E*. *triangularis* group. The authors explained how confusion occurred regarding *E. lunatus* (originally described from North Africa that belongs to the Palaearctic region) being erroneously included within the exclusively Afrotropical *Eumerus triangularis* group. This mistake most probably arose because specimens of *E. tessellatus* Hull, 1964 (a member of the *E. triangularis* group), were misidentified as *E. lunatus* by Doesburg, and Stackelberg [12] likely incorporated characters of *E. tessellatus* under the name *E. lunatus*, leading to erroneous interpretations of both morphology and distribution.
  Dirickx [91] listed *E*. *lunatus* for North Africa and cited an additional record from Linosa (Pelagic Islands, Italy) after Venturi [105] but noted that the species’ distribution was poorly known. The other European record, from the former Yugoslavia, listed by Glumac [106], was later rejected by Vujić & Šimić [107], who did not confirm the presence of *E*. *lunatus* after examination of available museum material.
  According to Speight [2], the only basis for including this species in the European list is a record by Venturi [105] from the island of Linosa (Italy) near North Africa. He also emphasised that this material should be located and re-examined, and suggested that subsequent descriptions of similar species, such as *E*. *etnensis*, should also be taken into consideration for resolving the status of *E*. *lunatus*. The connection between the two species is supported by the fact that the specimen from “Barbaria” belonging to the Fabricius collection deposited in the Berlin museum (ZMB) and wrongly labelled as “Type” of “*lunata*”, which refers to the subsequent transfer of the genus *Syrphus* to *Milesia* by Fabricius personally, belongs to *Eumerus etnensis*. Given the poor condition of the ZMUC holotype of *Eumerus lunatus* (with only part of the thorax and one wing preserved), the lack of verified European specimens, and repeated historical misidentifications, *E. lunatus* is here treated as a taxonomically uncertain species and is therefore not included in the European *Eumerus*; its final status remains to be fully resolved in ongoing research.

### 3.6. Species Richness and Endemism Patterns

The analysis of *Eumerus* diversity across European biogeographical regions reveals a distinct heterogeneity in both species richness (Figure 12) and the degree of local endemism. Total species richness was highest in the Mediterranean (MED) region (65 recorded species), followed by the Continental (CON) (25) and Alpine (ALP) (15) regions. However, when examining endemism, we focused exclusively on localised endemics (species restricted to specific islands, mountain ranges, or isolated habitats) rather than regional endemics (taxa widespread across an entire biogeographical province). Under this strict criterion, the Macaronesian (MAC) region exhibited the highest rate of endemism (75%), followed by the Mediterranean (28%) and the Pannonian (PAN) (25%) regions.

## 4. Discussion

The Mediterranean region stands out as the primary centre of diversity for the genus on the European continent, harbouring 65 species, which account for over 80% of the total European *Eumerus* fauna. This high concentration of taxa is likely the result of the region’s favourable climatic conditions, complex topography, and its role as a major Pleistocene refugium, which facilitated both the persistence of ancient lineages and rapid speciation [108,109]. These environmental drivers also fostered a high diversity of endemic forage and host plants, which in turn supported the radiation and specialisation of phytophagous lineages [18,52,110,111]. While inferring centres of origin solely from modern distributions requires caution given major past climatic shifts (including Holocene thermal fluctuations and glaciations), the region consistently exhibits high diversity and endemism.

This dominance is consistent with broader patterns observed in other phytophagous syrphids with similar life histories, most notably the genus *Merodon* Meigen, 1803, where the Mediterranean and Balkan refugia also support the vast majority of European species richness [112,113,114]. In contrast, the Continental and Boreal regions, while supporting 25 and 8 species, respectively, consist primarily of widespread, eurytopic taxa such as *Eumerus strigatus* and *E*. *funeralis*. The total absence of localised endemics in these northern and central regions suggests that their current *Eumerus* fauna is the result of post-glacial recolonisation rather than in situ speciation [115].

While absolute richness is highest in the Mediterranean, the Macaronesian region exhibits the highest rate of localised endemism at 75%, despite a lower total richness (12 recorded species, 9 of which are endemic). This reflects the geographic isolation of the Macaronesian archipelagos, where the restricted gene flow inherent to the Canary Islands and Madeira has driven unique speciation events [116,117]. The genus *Eumerus* represents a significant component of the Macaronesian syrphid fauna [48,117,118,119,120,121], comprising 20% of the total 65 recorded species belonging to 26 genera in the region according to Reverté et al. [122]. This is also reflected in the broader systematic structure of the European fauna; nearly all of the species unassigned to any of the 11 recognised species groups in Europe in our updated checklist are endemic to Macaronesia (*E. dubius*, *E. hispidus*, *E. latitarsis*, *E. macarius*, *E. nivariae*, and *E. purpureus*). The only exceptions to this pattern among the 10 unassigned species are *E. hungaricus* and *E. colladoi* and 2 newly recorded Oriental species (*E. aurifrons* and *E. figurans*).

Furthermore, the high endemism rate of the Pannonian region (25%) is a significant finding. Although relatively restricted in area, the unique alkaline steppes and sandy habitats of the Pannonian basin have fostered a specialised fauna, including species such as *E*. *pannonicus* and *E. banaticus* [13,123], both members of the predominantly endemic *bactrianus* group, which comprises mostly highly localised species. This identifies the Pannonian region as a critical continental centre of endemism within Europe [124,125].

From a systematic perspective, the composition of the updated checklist is heavily dominated by two species groups that comprise nearly half of the entire European fauna: the *tricolor* group (23 species, representing 28.4%) and the *strigatus* group (including the *strigatus/bactrianus* complex; 14 species, representing 17.3%). The high species richness within the *tricolor* lineage is particularly linked to the Mediterranean Basin, where localised speciation events have resulted in numerous highly restricted endemics (e.g., *E. ancylostylus*, *E. larvatus*, and *E. rubrum*). The *strigatus* group’s evolutionary success across diverse bioclimatic zones is likely tied to its larval host preferences, which favour widespread, bulbous geophytes.

The fauna of the Mediterranean has undergone significant changes in recent decades, driven by climate change and the increasing influx of species from tropical regions.

Sometimes, a single introduction of a non-native fly can have wide-ranging ecological consequences. For instance, the spotted-wing drosophila *Drosophila suzukii* (Matsumura, 1931) (*Diptera: Drosophilidae*), native to East Asia, was first recorded in Europe in 2008, almost simultaneously in coastal Spain and Tuscany, Italy, before rapidly invading the entire Mediterranean Basin and Central Europe [126,127,128,129]. This illustrates how dipteran introductions can trigger swift, widespread range expansions with substantial ecological and agricultural impacts.

The fauna of Central Europe has experienced significant changes in recent decades as well, primarily driven by climate change and the increasing influx of flora and fauna from tropical and Mediterranean regions. According to Haris et al. [130], there are 803 non-native insect species documented in Central Europe, with 298 of these introduced in the last 25 years. Notably, 54% of the recently introduced species hail from warmer climates, including 86 from the Mediterranean and 74 from tropical regions, which underscores a clear trend of biogeographical shifts.

Furthermore, human activities have significantly influenced the distribution and dispersal of insect species, particularly through the global translocation of host plants. The international trade of ornamental and agricultural plants has inadvertently facilitated the introduction of numerous insect species into non-native ecosystems, often leading to ecological and economic consequences. Among these, some hoverfly species have expanded their ranges due to human-mediated transportation, either through trade-related movement of their larval host plants or by utilising anthropogenic environments as new habitats. A notable example includes species whose life cycles are linked to plants, such as those from the genera *Eumerus* and *Merodon*. The larvae of these species feed on bulbs, tubers, stems, rhizomes, and/or decaying plant material. Some of the host plants are commercially marketed, which has led to an unintentional spread of hoverfly species to new regions. In this way, the *Merodon equestris* (Fabricius, 1794), *Eumerus funeralis*, and *E. narcissi*, all native to Europe, have been unintentionally introduced to Nearctic (*E. funeralis*, *E. narcissi*, and *E. strigatus*), Neotropics (*E. funeralis*) and Australasian regions (*E. funeralis, E. strigatus*, and *M*. *equestris*) through the trade of ornamental and agricultural plants, particularly *Narcissus*, *Lilium*, and *Allium* bulbs [9,16,28,29,30,31,51].

*Eumerus figurans* Walker, 1859, a species morphologically close to the *obliquus* group, classified by Ricarte et al. [8] within the *figurans* group, has been noted in Hawaii since 1900 [131,132] and was quite recently recorded in Paraguay [9]. This species, which also expanded its range from Southeast Asia through the Indian Ocean to Seychelles [20,100], is known to cause considerable damage to lily bulbs and ginger, making it an important agricultural pest; it has further bred out of decomposing pineapple stumps and taro roots [9,18,20,131,132,133,134,135,136] but is also an important pollinator [137]. This species is sometimes referred to as the “ginger fly”.

*Eumerus aurifrons* (Wiedemann, 1824), a species originally described from India, is widespread and commonly found in the Oriental region [9,51,76,77,78,80]. However, it is also present in other zoogeographical regions, such as the Australian [9,51,79] and the Afrotropical, covering most of the African continent, including Madagascar, as indicated in the unpublished key of Leif Lyneborg (1932–2006) and other publications [51,78]. Its distribution even reaches Hawaii and South America [9].

The larvae of *E*. *aurifrons* were found under the bark and in fermenting sap of the widespread *Albizia lebbeck* (Fabaceae) [138,139], which may have played a role in facilitating the range expansion of the species. Its potential host plant, *Albizia lebbeck*, originally native to India and Southeast Asia, has been introduced as an ornamental tree in various regions (South and North America, Africa including Madagascar, and the Middle East), including Sicily [140,141,142,143,144], where the first European record of *E*. *aurifrons* was documented.

In summary, the widespread cultivation of *Albizia lebbeck* as an ornamental plant has been established outside its native range for a long time [145], suggesting that the introduction of *Eumerus aurifrons* was mediated by human activity and plant trade, whereas its potential establishment and range expansion may be further facilitated by changing environmental conditions. Climate change, particularly rising temperatures and altered precipitation patterns, can create highly favourable microclimatic conditions for larval development, thereby serving as a critical catalyst for successful population establishment of introduced species, even if global warming itself was not the direct driver of the initial human-mediated introduction. The Mediterranean Basin is one of the most vulnerable regions to climate change, characterised by accelerated warming compared to the global average [146]. Within this context, Italy and its southern part, including Sicily, have experienced a significant rise in temperature, particularly in recent decades, together with an increasing frequency and intensity of extreme heat events [147]. This trend is exemplified by the record-breaking temperature of 48.8 °C recorded in Sicily on 11 August 2021, officially recognised as the highest temperature measured in Europe [148]. While sharing the same climate preferences with *E. aurifrons*, *E. figurans* possesses a significantly broader spectrum of larval host plants and is frequently transported alongside economically important crops such as ginger and lilies [20]. Species with more generalist feeding habits can more easily establish populations in new regions with suitable hosts. However, since these observations are based on only two records, further research and continuous monitoring are needed to better understand the species’ dispersal mechanisms and ecological interactions.

According to Speight [2], plus 2 additional species from Dirickx [91], 92 species of *Eumerus* are recognised in Europe. Excluding three species of Asian distribution (*E*. *anatolicus* Grković, Vujić, & Radenković, 2018, *E*. *hissaricus* Stackelberg, 1949, and *E*. *falsus* Becker, 1922), and accounting for three names now treated as synonyms (*E*. *bifurcatus* van Steenis & Hauser, 2019 = *E*. *pauper* Becker, 1921; *E*. *lateralis* (Zetterstedt, 1819) = *E*. *grandis* and *E*. *caballeroi* Gil Collado, 1929 = *E*. *nudus*), the number is reduced to 86 species.

Taking into account the recently described *Eumerus macarius* Ricarte & Aguado-Aranda, 2025, the four new synonymies proposed herein (*E*. *alpinus*, *E*. *aurofinis*, *E*. *lasiops*, and *E. nebrodesius*), four taxa (*E. acuticornis*, *E*. *angustifrons*, *E*. *graecus*, and *E*. *lunatus*) here treated as species of uncertain status and the addition of two species newly recorded for Europe (*E*. *aurifrons* and *E*. *figurans*), there is a total of 81 currently accepted European species of *Eumerus*.

According to The European Red List of Hoverflies [149], the genus *Eumerus* is of significant conservation concern in Europe: 43 of the 70 assessed species (over 60%) are classified in threatened categories, including 7 species assessed as Critically Endangered (CR). This pattern is not unique to *Eumerus*, as similarly high proportions of threatened species are recorded in other species-rich hoverfly genera, including *Merodon* (59%) and *Cheilosia* (40.5%).

Taking all the above into account, the genus *Eumerus* in Europe represents an important but often underestimated component of the pollinator fauna. Although it comprises a relatively small number of species compared to other Diptera groups and includes taxa that are occasionally associated with agricultural systems, its ecological role is complex and its species diversity considerable. Moreover, some species may have potential as bioindicators, as their occurrence and population dynamics can reflect habitat quality. Therefore, *Eumerus* constitutes a significant element of the European Diptera fauna and warrants particular attention in conservation planning, including reassessment of the conservation status of native European species and continued monitoring.

## Figures and Tables

**Figure 1:**
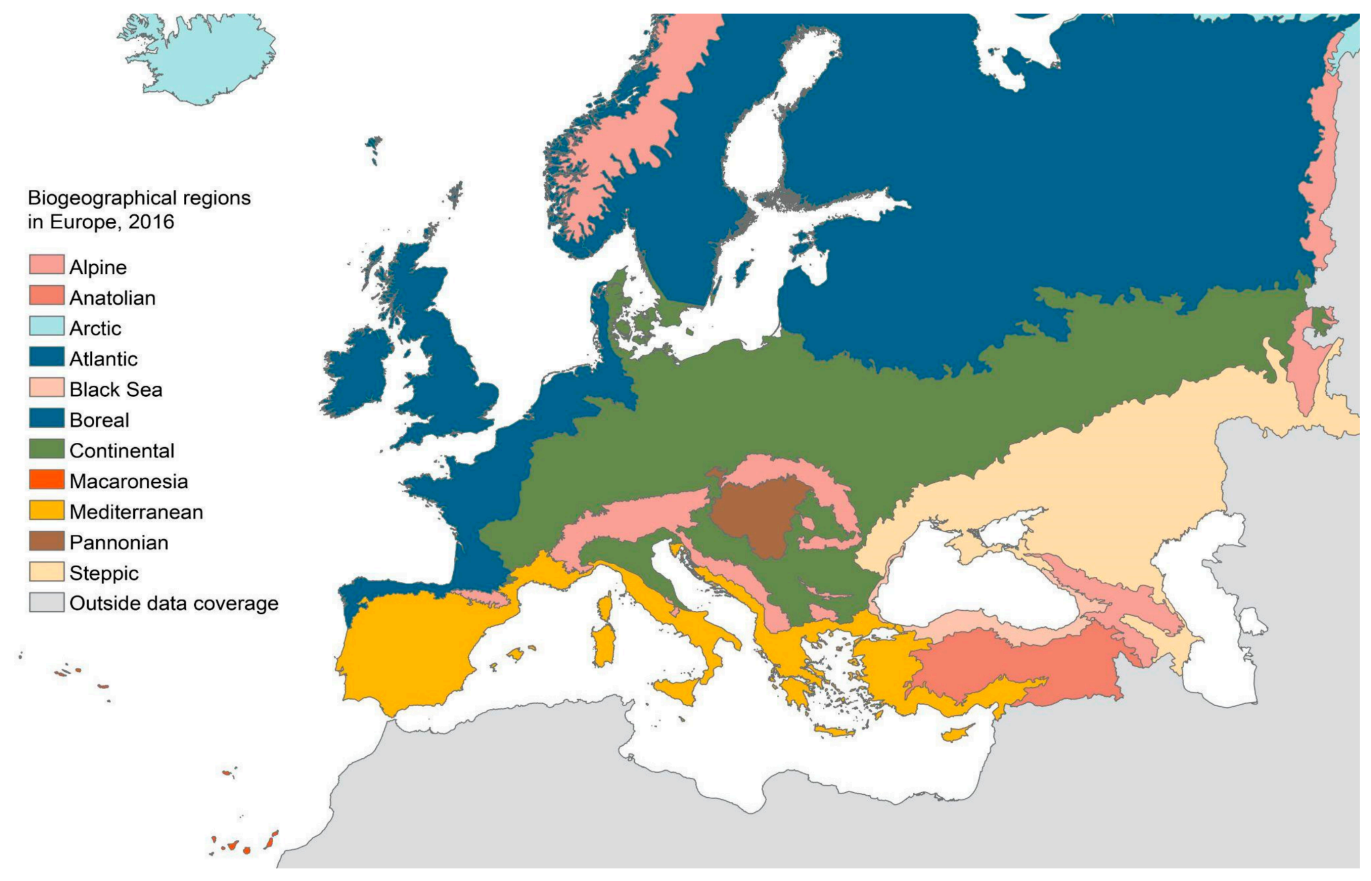
Biogeographical regions in Europe (EEA, 2017).

**Figure 12:**
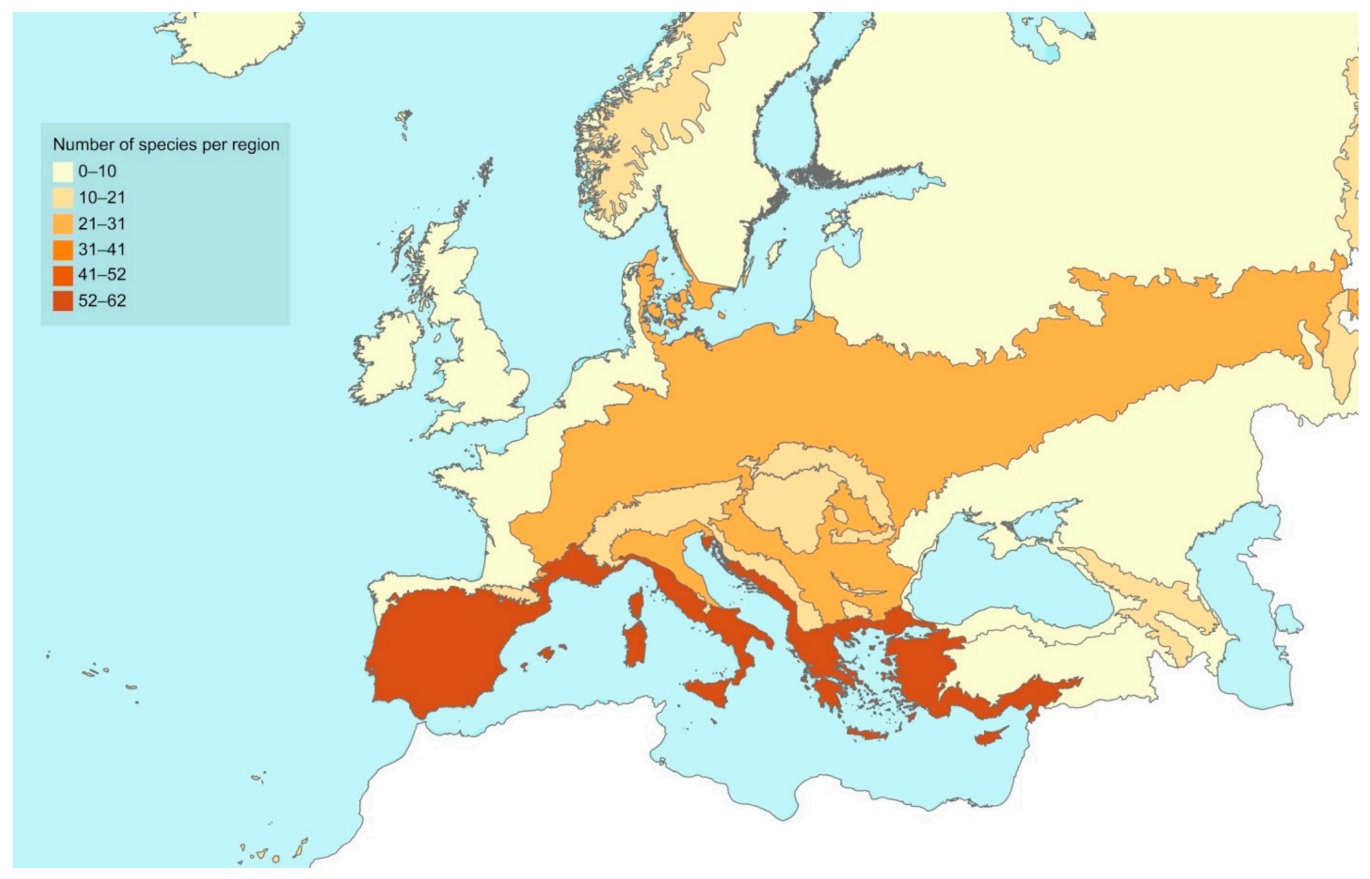
Species richness of the genus *Eumerus* Meigen, 1822 across the Biogeographical Regions of Europe (based on EEA 2017).

## Data Availability

The taxonomic checklist and regional richness summaries are available within the article. Occurrence data were sourced from iNaturalist, relevant literature, and the private collection of M.H. To protect sensitive collection data, precise locality records are available from the authors upon reasonable request.
